# Reduction of Calciprotein Particles in Adults Receiving Infliximab for Chronic Inflammatory Disease

**DOI:** 10.1002/jbm4.10497

**Published:** 2021-05-05

**Authors:** Mark K Tiong, Edward R Smith, Nigel D Toussaint, Hasan F Al‐Khayyat, Stephen G Holt

**Affiliations:** ^1^ Department of Nephrology The Royal Melbourne Hospital Parkville Australia; ^2^ Department of Medicine (RMH) University of Melbourne Parkville Australia; ^3^ SEHA Kidney Care Abu Dhabi Health Services Company Abu Dhabi United Arab Emirates; ^4^ Khalifa University Abu Dhabi United Arab Emirates

**Keywords:** BIOCHEMICAL MARKERS OF BONE TURNOVER, DISORDERS OF CALCIUM/PHOSPHATE METABOLISM, OSTEOIMMUNOLOGY, PTH/VIT D/FGF23

## Abstract

Patients with chronic inflammatory diseases (CID) experience accelerated loss of bone mineral density, which is often accompanied by increased vascular calcification. These disturbances can be attenuated by therapies for inflammation, such as the tumor necrosis factor inhibitor infliximab. Calciprotein particles (CPP) are circulating colloidal aggregates of calcium and phosphate together with the mineral‐binding protein fetuin‐A, which have emerged as potential mediators of vascular calcification. The precise origins of serum CPP are unclear, but bone turnover may be an important source. In this longitudinal observational study, we studied patients with CID undergoing treatment with infliximab to assess the temporal relationship between bone turnover and circulating CPP. Ten patients with active CID receiving infliximab induction therapy and an additional 3 patients with quiescent CID on maintenance infliximab therapy were studied for 8 weeks with repeated measures of bone turnover markers as well as CPP (calciprotein monomers [CPM], primary CPP [CPP‐I], and secondary CPP [CPP‐II]). Therapeutic response was appraised using validated disease activity scores. At baseline, those with active CID had elevated markers of bone resorption and suppressed bone formation markers as well as higher CPM and CPP‐I compared with those with quiescent CID. In responders, there was an early but transient reduction in resorption markers by week 1, but a more sustained increase in bone formation markers compared with non‐responders at week 8. This was accompanied by reductions in CPM (β = −6.5 × 10^3^ AU [95% CI −11.1, −1.8], *p* = 0.006) and CPP‐I (β = −23.4 × 10^4^ particles/mL [95% CI −34.8, −11.9], *p* < 0.001). In contrast, no significant changes in any markers were observed in non‐responders or those receiving maintenance therapy. Thus, CPP have a dynamic association with changes in bone turnover in response to infliximab therapy, adding to accumulating evidence of the role of bone as a determinant of systemic levels. © 2021 The Authors. *JBMR Plus* published by Wiley Periodicals LLC on behalf of American Society for Bone and Mineral Research.

## Introduction

A classic consequence of the aging process is a progressive loss of bone mineral content.^(^ ^1^ ^)^ The most prominent complication of this is a significant burden of fracture‐related morbidity and mortality.^(^ ^2^ ^)^ However, loss of bone mineralization is often also accompanied by a parallel, and paradoxical, increase in mineralization of blood vessels with an associated increased risk of cardiovascular disease (“the calcification paradox”).^(^ ^3^ ^)^ A propensity for accelerated bone and vascular disease is also found in several pathological conditions, including chronic kidney disease (CKD),^(^ ^4^ ^)^ as well as in chronic inflammatory diseases (CID) such as inflammatory bowel disease and inflammatory arthritis.^(^ ^5^, ^6^ ^)^

There is mounting evidence that bone loss and vascular calcification are directly linked through a series of interconnected pathways. Both processes share a number of common underlying risk factors, such as hypertension, diabetes, physical inactivity, smoking, excess alcohol consumption, as well as age and inflammation itself.^(^ ^7^ ^)^ In addition, newer insights into overlapping molecular pathways have strengthened the biological plausibility of a “bone‐vascular axis.”^(^ ^8^, ^9^ ^)^

An emerging pathway that has been implicated in vascular calcification is the formation of fetuin‐A‐containing calciprotein particles (CPP). Fetuin‐A is a hepatically derived plasma protein, which is a powerful inhibitor of ectopic mineralization.^(^ ^10^, ^11^ ^)^ In biological solutions, fetuin‐A is able to bind clusters of calcium and phosphate ions, forming calciprotein monomers (CPM). CPM may then self‐aggregate into 30‐ to 100‐nm amorphous primary calciprotein particles (CPP‐I), which may then transform into larger, denser 100‐ to 250‐nm secondary calciprotein particles (CPP‐II).^(^ ^12^ ^)^ This ordered formation of mineral‐protein colloids serves an important physiological role in facilitating bulk transport of mineral in circulation while preventing precipitation and ectopic deposition.^(^ ^13^ ^)^ However, in certain pathological conditions, including CKD and CID, elevated levels of circulating CPP are observed^(^ ^14^ ^)^ and have been positively associated with adverse events, including vascular stiffness,^(^ ^15^ ^)^ vascular calcification,^16^ ^)^ and mortality.^(^ ^17^ ^)^ In vitro studies have shown that CPP can induce vascular smooth muscle cell calcification and expression of inflammatory mediators,^(^ ^18^, ^19^ ^)^ while inhibiting osteoblast mineralization.^(^ ^20^ ^)^ Animal models have shown that circulating CPP can also induce vascular luminal^(^ ^21^ ^)^ and endothelial^(^ ^22^ ^)^ lesions. Taken together, it is proposed that elevated levels of CPP may not be just a marker of disordered mineral metabolism, but may themselves be mediators of vascular and bone pathology.

The precise origins of CPP in circulation and the drivers of elevated levels in pathological conditions are yet to be fully described. Previous animal^(^ ^23^ ^)^ and human^(^ ^24^, ^25^, ^26^ ^)^ studies have suggested that there may be some relationship with dietary intake. However, there is also a line of evidence to suggest that bone may be an important determinant of serum CPP. In early animal models, it was shown that CPP levels could be altered by manipulating bone turnover in rats with normal^(^ ^27^, ^28^ ^)^ and impaired^(^ ^29^ ^)^ kidney function. There are limited data exploring the link between bone and CPP in human studies. In patients with CKD, CPP levels have been observed to correlate with serum parathyroid hormone (PTH)^(^ ^30^ ^)^ and markers of bone turnover.^(^ ^15^, ^31^ ^)^ Beyond CKD, whether bone metabolism may also be linked to elevations in serum CPP in the setting of CID has not previously been explored in clinical studies.

Outcomes of patients with various forms of CID have been dramatically improved with the introduction of anti‐inflammatory and immunomodulatory biologic therapies such as tumor necrosis factor (TNF)‐alpha inhibitors, including infliximab. Exact indications are specific to individual conditions, but these agents are generally used in patients with moderate to high disease activity, often after failure to respond to conventional therapies. Their use in CID has been associated with improved bone mineral density and cardiovascular outcomes.^(^ ^32^, ^33^, ^34^, ^35^ ^)^

Bone loss in CID is driven by both suppressed bone formation and enhanced bone resorption, and TNF‐alpha inhibitor therapy has been shown to normalize markers of bone turnover.^(^ ^36^, ^37^ ^)^ Given the hypothetical links between CPP and bone turnover, we hypothesized that treatment of active CID with infliximab would lower circulating CPP levels and be temporally related to changes in markers of bone turnover.

## Materials and Methods

In this hypothesis‐generating, exploratory study, we recruited a cohort of patients with CID, who were planned to receive treatment with the TNF‐alpha inhibitor infliximab for active disease. This study was undertaken as part of the Fetuin‐A Levels in Systemic disease and Kidney Impairment (FLEKSI) study.^(^ ^14^ ^)^

We recruited a cohort of 10 patients with current active CID who were planned to receive induction therapy with infliximab. Participants had either inflammatory bowel disease (ulcerative colitis or Crohn's disease) or inflammatory arthritis (psoriatic arthritis or ankylosing spondylitis) and had disease‐specific indications for infliximab use (outlined in Table 1). For comparison, we also recruited an additional group of three patients with quiescent CID who were receiving maintenance therapy with infliximab (two with Crohn's disease and one with ulcerative colitis). Participants were recruited via the outpatient clinics at The Royal Melbourne Hospital. All participants were at least 18 years of age and gave written informed consent to participate in this study. Key exclusion criteria were participants who were unable to give informed consent or who were pregnant or breastfeeding. This study was approved by the local ethics committee (Melbourne Health Human Research Ethics Committee reference number MH 2012.141) and conducted in accordance with the Declaration of Helsinki.

**Table 1:** Baseline Demographics

	Overall (*n* = 13)	Induction therapy		Maintenance therapy
		Non‐responder (*n* = 4)	Responder (*n* = 6)	Quiescent (*n* = 3)
Age (years), mean (SD)	40.9 (11.8)	46.5 (15.4)	37.2 (11.9)	41 (4)
Sex—female, *n* (%)	7 (53.8)	4 (100)	2 (33.3)	1 (33.3)
Inflammatory arthritis, *n* (%)	3 (23.1)	1 (25)	2 (33.3)	–
Diagnosis and original indication for infliximab, *n* (%)				
Moderate to severe (Mayo score ≥ 6) active ulcerative colitis despite conventional therapy	4 (30.8)	1 (25)	2 (33.3)	1 (33.3)
Moderate to severe Crohn's disease (Crohn's Disease Activity Index score ≥ 220) despite conventional therapy	6 (46.2)	2 (50)	2 (33.3)	2 (66.7)
Active ankylosing spondylitis not responding to nonsteroidal anti‐inflammatory drugs	1 (7.7)	–	1 (16.7)	–
Severe active psoriatic arthritis not responding to conventional therapy	2 (15.4)	1 (25)	1 (16.7)	–

### Participant groupings and infliximab dosing

Patients with active disease received intravenous infliximab (Remicade, Janssen Immunology, Horsham, PA, USA) 5 mg/kg at 0, 2, and 6 weeks according to local dosing guidelines for induction therapy and in line with current international recommendations.^(^ ^38^ ^)^ The quiescent group received maintenance treatment with intravenous infliximab, at a dose of 5 mg/kg on an 8‐week schedule. Patients with active disease who received induction therapy were classified into “non‐responders” and “responders” based on their clinical and biochemical response to treatment. Criteria used for response was a decrease in Mayo score of ≥3 or a score of 0 for participants with ulcerative colitis; a decrease in Harvey‐Bradshaw Index ≥4 for participants with Crohn's disease; a decrease in Disease Activity Score‐28 ≥ 1.2 for participants with psoriatic arthritis; or a decrease in Ankylosing Spondylitis Disease Activity Score ≥ 1.1 for participants with ankylosing spondylitis.^(^ ^39^, ^40^, ^41^, ^42^, ^43^ ^)^ Assessment against these criteria were made by the participant's clinicians, who were otherwise not involved in the conduct of this study or analysis of results. These clinicians were also solely responsible for decisions regarding eligibility for and use of infliximab; however, each participant met local clinical eligibility criteria that are required for government funding of infliximab therapy^(^ ^44^ ^)^ and are consistent with international prescribing guidelines.^(^ ^45^ ^)^

### Variables

We performed serial measurements of the bone formation (osteoblast activity) markers bone‐specific alkaline phosphatase (bsALP) and procollagen type 1 N‐terminal propeptide (P1NP), bone resorption (osteoclast activity) markers tartrate‐resistant acid phosphatase 5b (TRAcP 5b) and C‐terminal telopeptide (CTx), CPP parameters (CPM, CPP‐I, CPP‐II), as well as intact and C‐terminal FGF23 (iFGF23 and cFGF23, respectively).

In the participants with active disease who were receiving induction therapy, we measured bone turnover markers (BTM), CPP, and FGF23 1 week before treatment as well as on the day of treatment and then on week 1, week 2, week 6, and week 8 after the initial dose of infliximab. To account for variation, the mean of values taken 1 week before and on the day of initial treatment was used as baseline (“week 0”) for our analysis. Participants receiving maintenance infliximab for quiescent disease had BTM, CPP, and FGF23 measured 1 week before and on the day of infliximab, as well as week 8 post dose. As for participants in the active group, for the quiescent group, we combined the values for the week before and the day of infliximab to calculate week 0. All participants also had blood collected at week 0 and week 8 for standard biochemistry as well as the cytokines interleukin (IL)‐6, ‐8, ‐10, ‐12, ‐17A, and ‐23, and fetuin‐A.

Venous blood samples were collected using standard phlebotomy procedures into serum, lithium heparin and K_2_‐EDTA tubes. Serum samples were allowed to clot at room temperature for 1h and then centrifuged at 2500g for 10 min. Plasma specimens were processed immediately following collection. Aliquots were stored at –80°C until batched analysis following a single thaw. Other biochemistries were performed by our institution in an accredited laboratory. Commercial immunoassay kits were used to measure serum IL (BioLegend, San Diego, CA, USA), serum fetuin‐A (R&D Systems, Minneapolis, MN, USA), BTM (Immunodiagnostic Systems, East Boldon, UK), and serum iFGF23 (Kainos Laboratories, Tokyo, Japan) and plasma cFGF23 (Immutopics Inc, San Clemente, CA, USA) according to the manufacturer's instructions. Plasma CPM was measured by gel‐filtration method as previously described.^(^ ^46^ ^)^ Serum CPP‐I and CPP‐II were measured using a fluorescent probe‐based assay running on a BD FACSVerse flow cytometer as previously described.^(^ ^23^, ^25^ ^)^

### Statistical analysis

Demographics and standard biochemical and cytokine data are presented using descriptive statistics. Mean and standard deviation or median and interquartile range are presented for continuous variables as appropriate, and number and percentage are presented for categorical variables.

Because there were no published data pertaining to the effect of infliximab on CPP or clinically important differences in levels, sample size calculations were based on our observation of a patient with Takayasu's arteritis,^(^ ^47^ ^)^ in whom infliximab treatment (5 mg/kg at 0 and 4 weeks) resulted in reduction in total CPP of 31% (25 × 10^4^/mL) from baseline to 8 weeks. We used GLIMMPSE, a validated linear mixed model power and sample size calculator,^(^ ^48^ ^)^ to conduct a priori sample size calculations. Assuming a mean baseline CPP‐I of 65 × 10^4^/mL and standard deviation of 8 × 10^4^/mL at each of the five time points, we estimated that a total of seven patients would be required to provide >90% power in detecting a linear decline in CPP‐I levels of 30% over 8 weeks at a 1% significance level (Geisser–Greenhouse corrected). Data for individual CPP components were not available to estimate appropriate sample sizes.

For patients with active disease receiving induction therapy with infliximab, we examined longitudinal between‐group differences for the non‐responder and responder groups for each measure of BTM, CPP, and FGF23. To do this while accounting for repeated measures, we fitted linear mixed‐effects models using a restricted maximum likelihood approach with an unstructured covariance structure. In each model response group, categorical time and time‐response group interaction were modeled as fixed effects, and we included a random intercept for each participant to allow for individual differences and correlation between repeated measures. In all these models, week 0 and non‐responder group were used as the reference levels for time and group, respectively. For the model of bsALP, we naturally log transformed values before fitting the model to ensure normal distribution of residuals. Estimates for bsALP were then back transformed for ease of interpretation. There was an apparent between‐group difference in the responder group compared with the non‐responder group in multiple BTM, CPP, and FGF23 variables. As a result, in further analyses, we fitted linear mixed effects models, restricted to data from the responder group, and using categorical time as the fixed effect, in order to confirm whether between‐group differences represented changes from baseline. Because this was an exploratory, hypothesis‐generating study, no correction for multiple testing was made. Two‐tailed *p* values <0.05 were considered significant. All data were analyzed using Stata version 16.1 (StataCorp, College Station, TX, USA) and figures were produced using GraphPad Prism version 9.0.0 (GraphPad Software, San Diego, CA, USA).

## Results

Of the 10 participants with active CID, 4 were adjudged by their treating clinician using disease‐specific criteria to be “non‐responders” and 6 to be “responders” after completing the induction regimen. The demographics of these participants, as well as the additional 3 patients with quiescent disease are presented in Table 1. The overall mean age of the cohort was 51 years and 54% were female. Three patients had inflammatory arthritis (2 in the responder group and 1 in the quiescent group), while the other 10 patients had inflammatory bowel disease.

Conventional biochemical measures as well as inflammatory markers at week 0 and week 8 are presented in Supplemental Table S1. All participants had normal kidney function at both time points. Groups appeared to have comparable levels of serum calcium, phosphate, albumin, bicarbonate, magnesium, total alkaline phosphatase, and PTH, while participants in the non‐responder group appeared to have lower levels of 25‐hydroxyvitamin D at both week 0 and week 8.

Expectedly, there were differences in the inflammatory marker C‐reactive protein (CRP) and interleukin panel between subgroups at baseline and over the 8‐week study period (Supplemental Table S1 and Supplemental Fig. S1). Participants with active disease tended to have higher levels of CRP and pro‐inflammatory cytokines at week 0 compared with those with quiescent disease. In the responder group, there were reductions in CRP, IL‐6, IL‐8, and IL‐17A from baseline to week 8, while there was an increase in the regulatory cytokine IL‐10. In contrast, levels of these markers appeared unchanged in non‐responders and those with quiescent disease. Consistent with fetuin‐A being a negative acute‐phase reactant, patients with active disease appeared to have lower levels of serum fetuin‐A than those with quiescent disease at baseline; however, levels appeared to rise in the responder group by week 8 (Supplemental Table S1).

### Bone turnover markers

**Fig 1:**
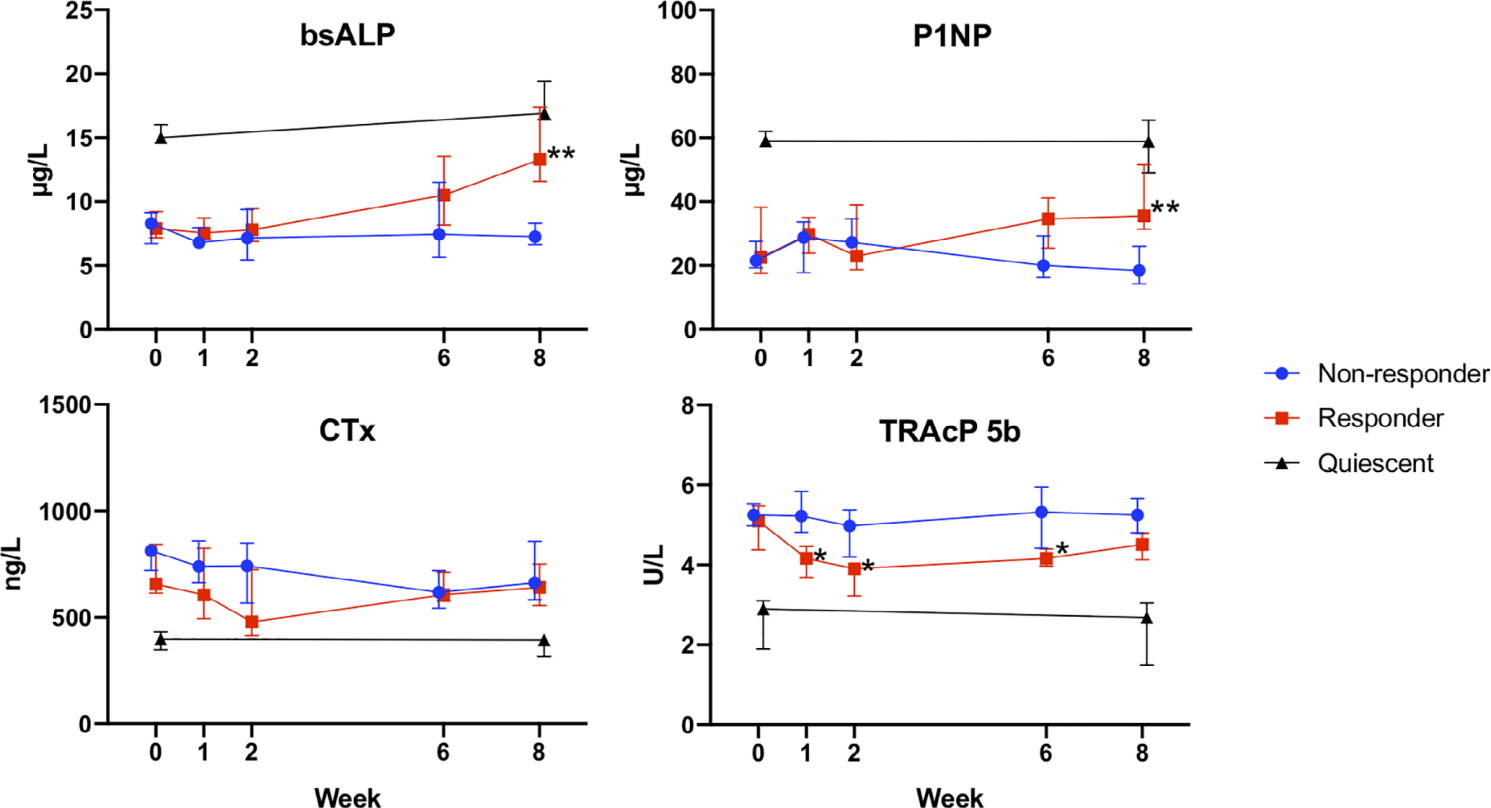
Change in bone turnover markers with infliximab treatment. Comparison between “non‐responder” and “responder” groups of bone formation (osteoblast) markers bsALP and P1NP as well as bone resorption (osteoclast) markers CTx and TRAcP 5b, during induction therapy with infliximab for active disease. Quiescent group also displayed for reference. Data presented as median with error bars showing interquartile range. Data points offset for clarity. *p < 0.05 for responder versus non‐responder, **p < 0.01 for responder versus non‐responder. bsALP = bone‐specific alkaline phosphatase; P1NP = procollagen type 1 N‐terminal propeptide; TRAcP 5b = tartrate‐resistant acid phosphatase 5b; CTx = C‐terminal telopeptide.

Repeated measures of BTM are displayed in Fig. 1 and Table 2. Coefficient estimates for linear mixed effects models fitted to the non‐responder and responder groups for each BTM are also presented in Table 2. At baseline, patients in the quiescent group appeared to have higher levels of the bone formation (osteoblast) markers bsALP and P1NP and lower levels of the bone resorption (osteoclast) markers CTx and TRAcP 5b than those with active disease.

**Table 2:** Linear Mixed‐Effect Models for Bone Turnover Markers in Patients With Active CID Receiving Infliximab Induction Therapy

	bsALP^a^	P1NP	CTx	TRAcP 5b
	μg/L	μg/L	ng/L	U/L
Group median (IQR)				
Non‐responder				
Baseline	8.3 (7.2, 9.0)	21.5 (19.5, 26.0)	813 (750, 824)	5.3 (5.1, 5.5)
Week 1	6.8 (6.6, 7.7)	28.8 (19.9, 33.6)	739 (686, 821)	5.2 (4.9, 5.7)
Week 2	7.2 (6.0, 8.7)	27.3 (26.4, 32.3)	742 (592, 847)	5.0 (4.5, 5.3)
Week 6	7.5 (5.9, 10.5)	20.0 (16.4, 27.4)	618 (567, 687)	5.3 (4.5, 6.0)
Week 8	7.3 (6.7, 8.2)	18.5 (15.6, 23.6)	664 (589, 814)	5.3 (4.9, 5.6)
Responder				
Baseline	7.9 (7.2, 8.3)	22.5 (20.0, 38.0)	657 (618, 839)	5.1 (4.4, 5.4)
Week 1	7.6 (7.2, 8.4)	29.8 (27.6, 34.5)	606 (522, 816)	4.2 (3.8, 4.4)
Week 2	7.8 (7.1, 9.3)	23.0 (20.4, 38.9)	480 (419, 680)	3.9 (3.4, 4.0)
Week 6	10.5 (8.5, 12.6)	34.7 (29.4, 40.5)	607 (584, 672)	4.2 (4.0, 4.4)
Week 8	13.3 (11.9, 17.3)	35.6 (33.8, 50.5)	640 (577, 738)	4.5 (4.2, 4.6)
Between‐group coefficient estimate^b^				
Week 1	0.5 (−1.8, 3.7)	−0.8 (−9.6, 7.9)	−36.8 (−231.9, 158.4)	−0.9* (−1.7, −0.2)
Week 2	0.6 (−1.7, 3.8)	−5.5 (−14.3, 3.2)	−84.1 (−279.4, 111.2)	−1.0* (−1.7, −0.2)
Week 6	2.4 (−0.4, 6.4)	8.2 (−0.6, 16.9)	97.6 (−97.9, 293.1)	−0.8* (−1.6, −0.1)
Week 8	6.4** (2.5, 11.9)	16.8** (8.1, 25.6)	27.7 (−168.1, 223.5)	−0.5 (−1.2, 0.3)
Coefficient estimate versus baseline for responder group only^c^				
Week 1	−0.5 (−1.9, 1.2)	3.2 (−1.5, 7.8)	−70.4 (−209.1, 68.3)	−0.9** (−1.4, −0.4)
Week 2	−0.3 (−1.8, 1.4)	1.1 (−3.6, 5.7)	−152.2* (−291.1, −13.4)	−1.3** (−1.8, −0.8)
Week 6	2.2* (0.4, 4.6)	7.3** (2.6, 12.0)	−62.8 (−201.8, 76.3)	−0.8** (−1.3, −0.3)
Week 8	5.5** (3.0, 8.5)	13.7** (9.0, 18.4)	−58.4 (−197.9, 81.1)	−0.5 (−1.0, 0.0)

CID = chronic inflammatory diseases; bsALP = bone‐specific alkaline phosphatase; P1NP = procollagen type 1 N‐terminal propeptide; CTx = C‐terminal telopeptide; TRAcP 5b = tartrate‐resistant acid phosphatase 5b; IQR = interquartile range.

Table shows group median (IQR) or mixed‐effects model coefficient (95% confidence interval).

Week 0 and non‐responder group used as reference groups for each model.

^a^ bsALP was naturally log transformed before fitting regression models to ensure normal distribution of residuals. Estimates have been back transformed for ease of interpretation.

^b^ Coefficient estimate is for responder group compared with non‐responder group.

^c^ Coefficient estimate is for categorical time compared with baseline for models fitted to responder group only.

* *p* < 0.05.

** *p* < 0.01.

In the responder group, a significant increase was observed at week 8 for both bsALP (β = 6.4 μg/L [95% confidence interval (CI) 2.5, 11.9], *p* < 0.001) and P1NP (β = 16.8 μg/L [95% CI 8.1, 25.6], *p* < 0.001), whereas TRAcP 5b showed a transient reduction in levels at week 1 (β = −0.93 U/L [95% CI −1.66, −0.19], *p* = 0.014), week 2 (β = −0.96 U/L [95% CI −1.70, −0.22], *p* = 0.011), and week 6 (β = −0.81 U/L [95% CI −1.55, −0.06], *p* = 0.011), but not at week 8 (β = −0.48 U/L [95% CI −1.23, 0.27], *p* = 0.207). In contrast, no change in CTx levels was detected at any time point in either group.

### Calciprotein particles

**Fig 2:**
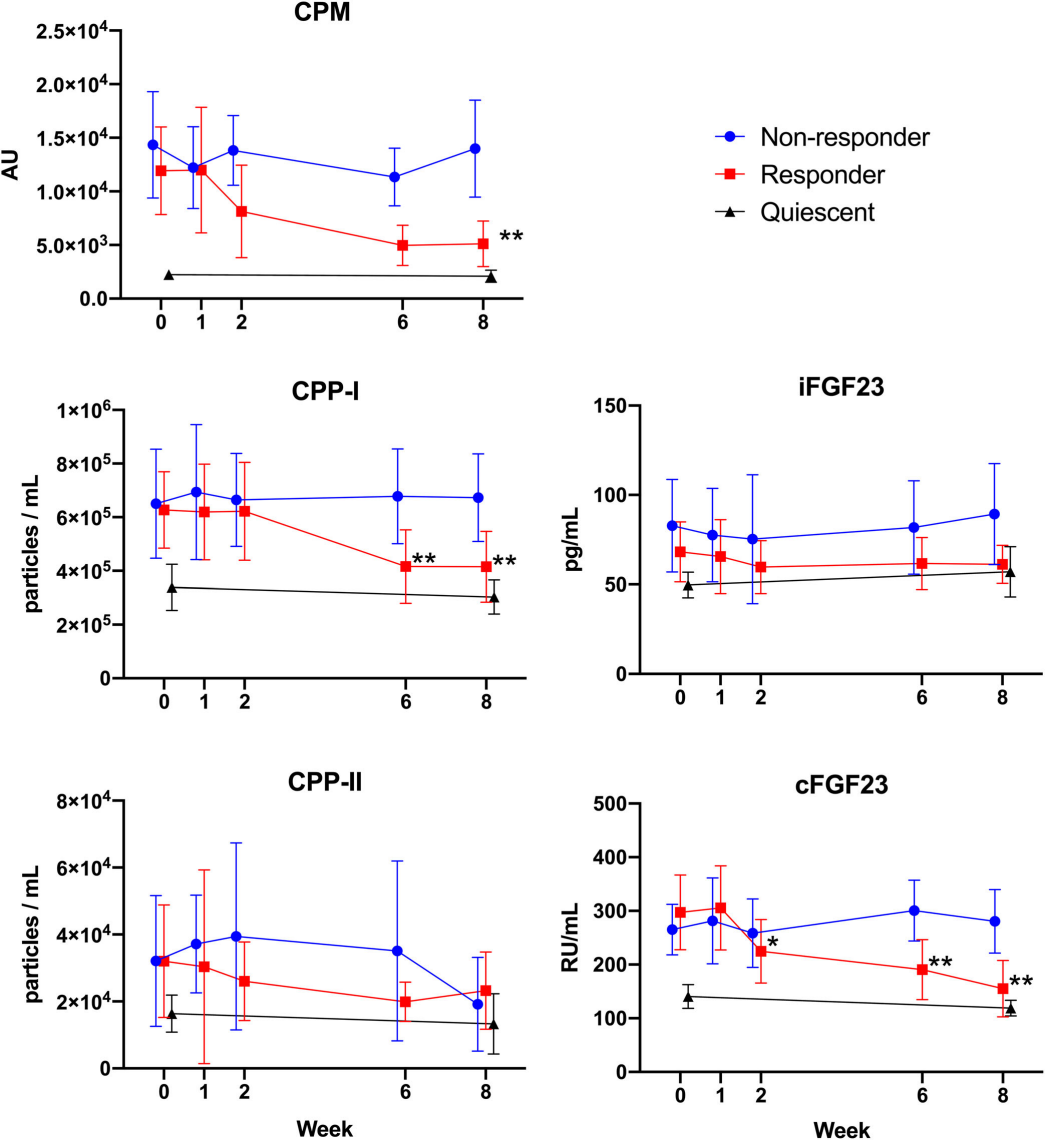
Change in calciprotein particles and fibroblast growth factor‐23 with infliximab treatment. Comparison between “non‐responder” and “responder” groups of CPM, CPP‐I, CPP‐II, iFGF23, and cFGF23 during induction therapy with infliximab for active disease. Quiescent group also displayed for reference. Normally distributed data (CPM, CPP‐I, and cFGF23) presented as mean with error bars showing standard deviation. Skewed data (CPP‐II and iFGF23) presented as median with error bars showing interquartile range. Data points offset for clarity. *p < 0.05 for responder versus non‐responder, ** *p* < 0.01 for responder versus non‐responder. CPM = calciprotein monomer; CPP = calciprotein particle; FGF23 = fibroblast growth factor‐23.

Participants in the quiescent group appeared to have lower levels of CPM and CPP‐I than the groups with active disease (Fig. 2 and Table 3). Coefficient estimates for linear mixed effects models fitted to the non‐responder and responder groups for CPP and FGF23 are presented in Table 3. In the responder group, there was a significant reduction in CPM at week 8 (β = −6.5 × 10^3^ AU [95% CI −11.1, −1.8], *p* = 0.006), while for CPP‐I, a significant reduction was evident at week 6 (β = −23.8 × 10^4^ particles/mL [95% CI −34.7, −12.8], *p* < 0.001) and was sustained at week 8 (β = −23.4 × 10^4^ particles/mL [95% CI −34.8, −11.9], *p* < 0.001).

**Table 3:** Linear Mixed‐Effect Models for Calciprotein Particles and Fibroblast Growth Factor‐23 in Patients With Active CID Receiving Infliximab Induction Therapy

	CPM	CPP‐I	CPP‐II	iFGF23	cFGF23
	×10^3^ AU	×10^4^/mL	×10^3^/mL	pg/mL	RU/mL
Group mean (SD) or median (IQR)					
Non‐responder					
Baseline	14.3 (5.0)	65.1 (20.4)	33.3 (15.8, 48.4)	85.0 (64.0, 101.6)	265.2 (47.2)
Week 1	12.2 (3.8)	69.4 (25.1)	36.7 (24.8, 49.5)	79.2 (58.0, 97.0)	281.4 (80.2)
Week 2	13.8 (3.3)	66.4 (17.3)	39.7 (16.5, 62.3)	69.1 (49.9, 100.7)	258.5 (63.7)
Week 6	11.3 (2.7)	67.8 (17.7)	30.5 (14.0, 56.2)	83.3 (64.9, 98.5)	300.3 (56.5)
Week 8	14.0 (4.5)	67.3 (16.3)	13.7 (10.9, 27.4)	90.7 (69.1, 109.5)	280.4 (58.8)
Responder					
Baseline	11.9 (4.1)	62.7 (14.2)	30.3 (15.2, 43.3)	70.7 (59.1, 77.2)	297.4 (69.7)
Week 1	12.0 (5.9)	61.9 (17.8)	16.2 (9.2, 52.0)	63.7 (51.0, 88.4)	305.8 (78.6)
Week 2	8.1 (4.3)	62.2 (18.2)	29.2 (15.0, 34.8)	54.3 (52.6, 74.7)	224.6 (59.2)
Week 6	5.0 (1.9)	41.5 (13.7)	17.4 (15.4, 26.3)	61.6 (48.2, 71.4)	190.5 (56.1)
Week 8	5.1 (2.1)	41.5 (13.1)	24.7 (15.9, 30.3)	61.4 (57.9, 65.4)	155.2 (52.4)
Between‐group coefficient estimate^a^					
Week 1	2.2 (−2.5, 6.8)	−5.1 (−15.5, 5.3)	−6.7 (−38.4, 24.9)	2.7 (−13.5, 18.9)	−7.9 (−70.9, 55.2)
Week 2	−3.3 (−7.9, 1.4)	−1.9 (−12.5, 8.8)	−13.3 (−45.2, 18.5)	−1.2 (−18.0, 15.5)	−66.1* (−130.5, −1.7)
Week 6	−4.0 (−8.6, 0.7)	−23.8** (−34.7, −12.8)	−15.2 (−47.3, 17.0)	−5.3 (−23.0, 12.4)	−142.0** (−208.6, −75.4)
Week 8	−6.5** (−11.1, −1.8)	−23.4** (−34.8, −11.9)	4.2 (−28.5, 36.8)	−13.6 (−32.6, 5.3)	−157.4** (−227.0, −87.8)
Coefficient estimate versus baseline for responder group only^b^					
Week 1	0.1 (−3.0, 3.1)	−0.8 (−8.0, 6.5)	−1.7 (−18.0, 14.6)	−2.6 (−13.0, 7.8)	8.4 (−36.1, 52.8)
Week 2	−3.8* (−7.0, −0.6)	−0.5 (−7.8, 6.8)	−6.0 (−22.8, 10.8)	−8.8 (−20.1, 2.6)	−72.9** (−118.6, −27.1)
Week 6	−7.0** (−10.5, −3.4)	−21.1** (−28.4, −13.7)	−12.2 (−29.8, 5.5)	−6.5 (−19.2, 6.3)	−106.9** (−154.8, −59.1)
Week 8	−6.8** (−10.7, −2.9)	−21.2** (−28.6, −13.8)	−8.8 (−27.6, 10.0)	−7.2 (−21.7, 7.4)	−142.2** (−192.8, −91.6)

CID = chronic inflammatory diseases; CPM = calciprotein monomer; CPP = calciprotein particle; cFGF23 = c‐terminal fibroblast gorwth factor 23; iFGF23 = intact fibroblast growth factor‐23; SD = standard deviation; IQR = interquartile range.

Table shows group mean (SD), median (IQR), or mixed‐effects model coefficient (95% confidence interval).

Week 0 and non‐responder group used as reference groups for each model.

^a^ Coefficient estimate is for responder group compared with non‐responder group.

^b^ Coefficient estimate is for categorical time compared with baseline for models fitted to responder group only.

^*^ *p* < 0.05.

^**^ *p* < 0.01.

For CPP‐II, there was no clear difference between those with active and quiescent disease at baseline or serially, and no between‐group difference was observed at week 8 (β = 4.2 × 10^3^ particles/mL [95% CI −2.9, 3.7], *p* = 0.803).

### Fibroblast growth factor‐23

The quiescent group appeared to have lower levels of cFGF23 than the non‐responder and responder groups at baseline (Fig. 2 and Table 3). Longitudinally, a significant reduction in cFGF23 was observed for the responder group, which was evident by week 2 (β = −66.1 RU/mL [95% CI −130.5, −1.7], *p* = 0.044) and persisted at week 6 (β = −142.0 RU/mL [95% CI −208.6, −75.4], *p* < 0.001) and at week 8 (β = −157.4 RU/mL [95% CI −227.0, −87.8], *p* < 0.001). In contrast, no significant difference in iFGF23 between the non‐responder and responder groups were observed at any time point.

### Linear mixed‐effect models restricted to responder group

Given the between‐group differences found in several of the above variables, linear mixed effect models were fitted to data restricted to the responder group to confirm whether between‐group differences also represented change from baseline values (Tables 2 and 3). These analyses confirmed that there was a significant increase at week 8 for bsALP (β = 5.5 μg/L [95% CI 3.0, 8.5], *p* < 0.001) and P1NP (β = 13.7 μg/L [95% CI 9.0, 18.4], *p* < 0.001) compared with baseline. There were also significant decreases for CPM (β = −6.8 × 10^3^ particles/mL [95% CI −10.7, −2.9], *p* = 0.001), CPP‐I (β = −21.2 × 10^4^ particles/mL [95% CI −28.6, −13.8], *p* < 0.001), and cFGF23 (β = −142.2 RU/mL [95% CI −192.8, −91.6], *p* < 0.001) at week 8 compared with baseline.

## Discussion

In this pilot, hypothesis‐generating observational study, our data suggest that modulation of inflammation in patients with CID is associated with dynamic changes in bone turnover profile and that this is paralleled by changes in CPP and FGF23 metabolism. To our knowledge, this is the first study to report that interventions with anti‐inflammatory and immunomodulatory anti‐TNF biologics like infliximab might be useful in modifying CPP levels. At baseline, we found that patients with active inflammation had evidence of suppressed bone formation (osteoblast activity) and high bone resorption (osteoclast activity), which was accompanied by high levels of CPM, CPP‐I, and cFGF23. Serially, patients who showed response to infliximab demonstrated improvements in bone formation markers, which were accompanied by reductions in CPM, CPP‐I, and cFGF23. Notably, all these longitudinal changes appeared to trend significantly toward values observed in the quiescent group.

Our data add to the accumulating evidence that alterations in bone metabolism may be a significant determinant of CPP and its precursors (CPM) in the circulation. While at baseline, elevated levels of CPM and CPP‐I were observed alongside both suppressed bone formation and high bone resorption, it is notable that only effects on bsALP and P1NP appeared sustained at week 8 in responders, whereas changes in the osteoclast marker TRAcP 5b occurred much earlier, were transient, and not mirrored by changes in CTx. Indeed, the increase in bone formation markers appeared to more closely parallel reductions in CPM and CPP‐I. Given these observations, we speculate that bone formation may be the dominant driver underlying serum levels of CPP in CID. This contrasts with observations in dialysis cohorts, where CPP positively correlate with the degree of hyperparathyroidism, a condition where high turnover bone disease and enhanced osteoclastic activity is a prominent feature.^(^ ^30^ ^)^ Moreover, in a post hoc analysis of a randomized trial of hemodialysis patients comparing standard to high dialysate magnesium, Bressendorff and colleagues found that while CPP were weakly negatively correlated with bsALP, the association with TRAcP 5b was nearly twice as strong.^(^ ^31^ ^)^ Taken together, this suggests both formation and resorption components of remodeling may contribute to changes in circulating CPP and it is the net balance or coupling of these processes that determines effects on the serum CPP pool. Further work is needed to validate these findings against gold standard assessment of bone turnover by histomorphometric analysis of bone biopsies.

Compatible with these observations in humans, early animal models by Price and colleagues found that use of etidronate at supratherapeutic doses known to acutely inhibit bone formation was associated with the appearance of CPP in circulation.^(^ ^27^ ^)^ In subsequent work, they found that this effect was ameliorated when the animals were first pretreated with various inhibitors of bone resorption.^(^ ^28^ ^)^ A notable point of difference between these experiments and a vailable clinical data is that the supratherapeutic doses of etidronate also led to significant fluctuations in serum calcium and phosphate and potentially resulting in intravascular CPP formation. Nevertheless, one plausible explanation of this experimental data, as well as previous clinical studies in CKD and now in CID, is that bone acts as both a source and a sink of CPP or its precursors.^(^ ^23^ ^)^ Therefore, it may be that in situations where rates of bone formation and bone resorption are geared toward accelerated net bone loss, this leads to elevated serum CPP, especially when mineral excretion is impaired as in CKD.

The hypothesis eluded to here is still lacking a complete mechanistic explanation of how alterations in bone turnover would eventually result in elevations of CPP in circulation. In lab‐based experiments, under physiological conditions, the process from assembly of CPM, which coalesce to form CPP‐I and ultimately CPP‐II, takes hours.^(^ ^49^ ^)^ By comparison, injected artificial CPP particles are rapidly cleared from circulation within minutes,^(^ ^19^ ^)^ and so doubts have previously been raised whether CPP could be assembled in significant numbers within the serum itself.^(^ ^49^ ^)^ Additionally, a “bone‐centric” paradigm of CPP formation also does not exclude the gastrointestinal tract from being an important contributor. Previous studies in animals^(^ ^23^ ^)^ and dialysis patients^(^ ^25^, ^26^ ^)^ have shown that serum CPP can be manipulated through the gastrointestinal (GI) tract, and it is possible that an interrelationship between the gastrointestinal tract, bone, and CPP may exist.^(^ ^12^ ^)^ Indeed, given that the majority of patients in this pilot study had GI disease, it is plausible that improvement in GI tract barrier function in response to infliximab may have imparted effects on the absorption of mineral.^(^ ^50^ ^)^

The clinical interest in CPP stems mostly from its apparent role as a mediator of vascular toxicity. However, almost all of the human data linking CPP to clinically relevant events come from patients with CKD. While CPP have also been found to be elevated in CID, larger studies incorporating vascular, patient relevant outcomes are needed to clarify the significance of CPP in this setting. While we found a reduction of CPM and CPP‐I with treatment of the underlying inflammatory condition, there was less clear delineation for CPP‐II between those with active and quiescent disease at baseline and no clear longitudinal change between response groups. This is potentially of significance, given that several preclinical studies have suggested that CPP‐II is the main mediator of cellular toxicity, albeit more recent preclinical,^(^ ^19^ ^)^ and clinical data have suggested that smaller CPP species are also associated with important outcomes.^(^ ^16^, ^17^ ^)^ Our failure to detect a change in CPP‐II may reflect our small sample size; however, it is notable that some,^(^ ^25^, ^30^ ^)^ but not all,^(^ ^31^ ^)^ previous clinical studies using the same flow cytometric assay also found that only CPP‐I was responsive to intervention. Given the potential link between CPP and bone, if CPP is also confirmed to be associated with vascular outcomes in CID, then this may suggest a role of CPP in the “bone‐vascular axis.” Importantly, given the reductions of CPM and CPP‐I with treatment, it is possible that CPP could act as not only a marker but also a modifiable risk factor for bone and/or vascular outcomes, through therapeutic interventions targeting drivers of high bone turnover, including systemic inflammation. Clearly, further work is needed to clarify the mechanistic role, and ultimately, the clinical utility of CPP measured in these processes.

Fetuin‐A, an indispensable component of CPP as well as its precursors, has notable links to inflammation, as well as bone and mineral metabolism. Fetuin‐A is considered a negative acute‐phase reactant,^(^ ^51^ ^)^ and consistent with this, we observed lower serum levels in those with active disease, which numerically increased in responders after induction therapy with infliximab. Although primarily secreted by hepatocytes in adults, fetuin‐A extravasates from extracellular fluid to mineralized tissues because of its high affinity for hydroxyapatite and in bone represents one of the most abundant of the non‐collagenous proteins.^(^ ^52^ ^)^ Fetuin‐A has also previously been associated with significant clinical outcomes, with serum levels showing inverse associations with vascular calcification,^(^ ^53^ ^)^ aortic stiffness,^(^ ^54^ ^)^ and mortality.^(^ ^55^ ^)^ Nevertheless, these associations have not been uniform,^(^ ^56^ ^)^ and one explanation for this variability is that the mineral‐bound fetuin‐A (ie, CPP) may be the more relevant metric of pathology than total serum levels.^(^ ^57^ ^)^

The peptide hormone FGF23 is another factor with evidence of biological toxicity and with intriguing links to inflammation and bone.^(^ ^58^ ^)^ FGF23 is itself bone‐derived and primarily functions as an endocrine regulator of phosphate excretion and vitamin D metabolism in the kidney.^(^ ^59^ ^)^ In CKD populations, it has consistently been positively associated with cardiovascular events and death, although whether this is through direct causation is unclear.^(^ ^60^ ^)^ While the exact mechanism(s) regulating FGF23 in health and disease remain under investigation, levels have previously been shown to positively correlate with inflammation, including in clinical studies of patients with rheumatoid arthritis and inflammatory bowel disease.^(^ ^61^, ^62^ ^)^ FGF23 has also been observed to correlate with CPP,^(^ ^23^, ^24^ ^)^ and recently, CPP have been proposed to be a direct mediator of FGF23 expression in bone cells.^(^ ^63^ ^)^

Inactivation of FGF23 by posttranslational cleavage is believed to be an important step in the regulation of FGF23 bioactivity,^(^ ^64^ ^)^ and this can be captured ex vivo by measurement of FGF23 in blood using intact and C‐terminal assays in tandem. Whereas intact FGF23 assays exclusively measure the full‐length protein, the C‐terminal assay detects both the full‐length hormone and cleaved C‐terminal fragments. In this study, the pattern of changes in cFGF23 largely followed that of CPM and CPP‐I, with patients with active CID demonstrating higher levels than the quiescent group at baseline, and those responding to infliximab therapy showing progressive reductions in levels over 8 weeks. By comparison, we did not find evidence of differences in intact FGF23 at baseline or longitudinally. Interestingly, pro‐inflammatory stimuli have been found to differentially effect intact and C‐terminal FGF23 concentrations in osteocyte cultures and mouse models.^(^ ^65^, ^66^ ^)^ Thus it is possible that inflammation, and potentially CPP, might drive an increase FGF23 transcription in CID, but that this is accompanied by an appropriate and compensatory upregulation of cleavage to maintain overall bioactivity and null effects on phosphaturia and vitamin D. This could explain the elevation in cFGF23 in patients with active inflammation at baseline which “normalized” in responders, while also accounting for the lack of signal in iFGF23.

Our study has a number of limitations. We studied a small and heterogenous group of patients. Inflammatory bowel disease and inflammatory arthritis each encompass diverse disease entities with distinct pathogenesis and clinical manifestations. Nonetheless, these conditions share a number of common features, including an apparent environmental stimulus, often in individuals with genetic susceptibility, triggering an exaggerated and sustained inflammatory response. Indeed, most CIDs also share a propensity for accelerated systemic bone loss and vascular calcification. Importantly, another shared feature of these disease entities is the efficacy of treatment with TNF‐alpha inhibitors, such as infliximab, supporting the idea of shared pathological pathways.

Given the small sample size, we did not undertake formal comparison testing of between‐group demographics, nor did we attempt to make adjustments for any demographic variable in our models. For similar reasons, we also considered it inappropriate to formally test for correlations between variables or to include the quiescent group in our linear mixed models, as this would have significantly added to the number of comparisons applied to our limited number of participants. Hence, formal hypothesis testing was limited to a small number of predefined outcomes relating to CPP and bone markers parameters. This was always intended as a hypothesis‐generating study, and larger trials are needed to test the robustness of the apparent associations that we describe. Nevertheless, considering our small heterogenous cohort, the delineation between patients with active disease and quiescent disease in several of the study variables at baseline was remarkable. Further, the tendency of multiple variables in the responder group to trend toward values found in the quiescent group was also impressive in their uniformity. Exceptions to this were the bone resorption markers CTx and TRAcP 5b. However, considering the small numbers and relatively short observational period, it is possible that TRAcP 5b and/or CTx may “normalize” in larger or longer‐term studies, as has previously been suggested.^(^ ^36^, ^37^ ^)^

Given the observational nature of this study, our findings are associative, and we can only speculate on potential underlying mechanistic links. Our notion of CPP being directly linked to bone turnover requires confirmation in future carefully designed studies. In particular, we speculate that inflammation drives dynamic changes in bone turnover, which sequentially results in elevations in CPP. These assumptions are largely based on the well‐described effects of inflammation on bone turnover, driven by the cross‐talk between multiple cytokine and bone cell signaling pathways.^(^ ^67^ ^)^ Given that our observations are temporarily linked to the administration of infliximab, it seems reasonable to assume that our observations of CPP are “downstream” to the effects of inflammation modulation and bone turnover. However, it is notable that in lab studies, CPP itself has been found to induce cellular inflammatory responses and also to inhibit osteoblast mineralization.^(^ ^18^, ^19^, ^20^ ^)^ As such, if interactions between inflammation, bone turnover, and CPP are confirmed, they may not necessarily be unidirectional. Similarly, both inflammation^(^ ^65^ ^)^ and CPP^(^ ^63^ ^)^ have previously been proposed as modulators of FGF23 expression, and our data do not allow us to clarify the hierarchy of these effects.

In conclusion, our data suggest that infliximab induction therapy is associated with an increase in bone formation markers and concomitant reductions in CPM and CPP‐I in patients with active CID. This raises the possibility that bone turnover may be a significant determinant of serum CPP levels and that in situations of net bone loss commonly found in CID and CKD may lead to expansion of the circulating CPP pool. Given the growing evidence implicating CPP as a mediator of vascular disease, this may also suggest that elevations in CPP form part of the disturbed bone‐vascular axis in CID.

## Disclosures

MKT reports no disclosures. ERS holds stock in Calciscon and has received grant support from Amgen, Baxter, and Sanofi. NDT reports research funding and honoraria from Amgen, Sanofi, and Shire Pharmaceuticals. HFA‐K reports no disclosures. SGH reports research funding from Amgen, Baxter, and Sanofi.

### Peer review

The peer review history for this article is available at https://publons.com/publon/10.1002/jbm4.10497.

## Supporting information


**Supplemental Table S1.** Biochemical and Interleukin Profile at Week 0 and Week 8 by Subgroup
**Supplemental Fig. S1.** C‐Reactive Protein (CRP) and Interleukin (IL) Panel at Baseline and Week 8 by Subgroup
