# Supplementary material for: Reduction of Calciprotein Particles in Adults Receiving Infliximab for Chronic Inflammatory Disease
Source: JBMR Plus. 2021 May 5;5(6):e10497. doi: 10.1002/jbm4.10497 (PMC8216135; doi:10.1002/jbm4.10497)
Supplement: Supplementary file 1 — Supplemental Table S1. Biochemical and Interleukin Profile at Week 0 and Week 8 by Subgroup Supplemental Fig. S1. C‐Reactive Protein (CRP) and Interleukin (IL) Panel at Baseline and Week 8 by Subgroup [file JBM4-5-e10497-s001.docx]

**Supplemental Data**

**REDUCTION OF CALCIPROTEIN PARTICLES IN ADULTS RECEIVING INFLIXIMAB FOR CHRONIC INFLAMMATORY DISEASE**

Mark K. TIONG^1,2^, Edward R. SMITH^1,2^, Nigel D. TOUSSAINT^1,2^, Hasan F. AL-KHAYYAT^1^, Stephen G. HOLT^1,2,3,4^

*^1^Department of Nephrology, The Royal Melbourne Hospital, Parkville, Australia;*

*^2^Department of Medicine (RMH), University of Melbourne, Parkville, Australia;*

*^3^SEHA Kidney Care, Abu Dhabi Health Services Company, Abu Dhabi, United Arab Emirates;*

*^4^Khalifa University, Abu Dhabi, United Arab Emirates.*

**Supplemental Table 1:** Biochemical and interleukin profile at Week 0 and Week 8 by subgroup

|  | **Non Responder**  **(n=4)** | **Responder**  **(n=6)** | **Quiescent**  **(n=3)** |
| --- | --- | --- | --- |
| Creatinine (µmol/L) |  |  |  |
| Week 0 | 66.0 (8.3) | 67.2 (6.4) | - 1. (5.1) |
| Week 8 | 69.8 (6.6) | 65.7 (3.9) | 69.9 (3.5) |
| Calcium (mmol/L) |  |  |  |
| Week 0 | 2.28 (0.05) | 2.41 (0.11) | 2.23 (0.01) |
| Week 8 | 2.32 (0.10) | 2.36 (0.07) | 2.26 (0.05) |
| Phosphate (mmol/L) |  |  |  |
| Week 0 | 0.81 (0.14) | 0.83 (0.24) | - 1. (0.25) |
| Week 8 | 0.77 (0.23) | 0.93 (0.23) | 0.92 (0.24) |
| Albumin (g/L) |  |  |  |
| Week 0 | 37 (34, 38) | 38 (37, 41) | 40 (39, 41) |
| Week 8 | 37 (34, 39) | 39 (37, 40) | 41 (39, 42) |
| Bicarbonate (mmol/L) |  |  |  |
| Week 0 | 26.3 (1.0) | 26.0 (1.8) | - 1. (1.2) |
| Week 8 | 27.5 (0.6) | 27.3 (1.9) | 25.3 (0.6) |
| Magnesium (mmol/L) |  |  |  |
| Week 0 | 0.78 (0.13) | 0.79 (0.06) | 0.82 (0.07) |
| Week 8 | 0.73 (0.05) | 0.79 (0.10) | 0.87 (0.07) |
| ALP (IU/L) |  |  |  |
| Week 0 | 55.8 (20.6) | 65.5 (17.0) | 1. (11.4) |
| Week 8 | 51.3 (12.6) | 68.8 (16.9) | 57.0 (7.5) |
| 25-OH vitamin D (nmol/L) |  |  |  |
| Week 0 | 34.5 (18.7) | 62.3 (16.9) | 1. (24.3) |
| Week 8 | 36.5 (13.3) | 68.8 (22.1) | 71.7 (15.9) |
| PTH (pmol/L) |  |  |  |
| Week 0 | 6.0 (3.5, 8.4) | 4.4 (4.0, 5.5) | - 1. (3.8, 4.9) |
| Week 8 | 5.9 (3.7, 8.3) | 4.1 (3.5, 5.7) | 5.6 (4.4, 5.7) |
| CRP (mg/L) |  |  |  |
| Week 0 | 26.2 (17.8, 33.4) | 25.2 (22.9, 27.1) | - 1. (0.3, 0.7) |
| Week 8 | 21.8 (16.4, 26.8) | 6.8 (4.7, 10.4) | 0.9 (0.4, 1.4) |
| Fetuin-A (g/L) |  |  |  |
| Week 0 | 0.39 (0.24) | 0.35 (0.14) | 0.64 (0.11) |
| Week 8 | 0.41 (0.21) | 0.52 (0.09) | 0.66 (0.15) |
| IL-6 (pg/mL) |  |  |  |
| Week 0 | 36.1 (14.9) | 33.8 (17.3) | 3.3 (1.5) |
| Week 8 | 48.2 (19.1) | 16.9 (8.3) | 4.6 (2.4) |
| IL-8 (pg/mL) |  |  |  |
| Week 0 | 34.7 (31.5, 57.5) | 33.9 (25.5, 56.7) | - 1. (3.9, 5.7) |
| Week 8 | 59.0 (40.6, 92.3) | 15.0 (9.1, 19.8) | 2.8 (2.0, 7.5) |
| IL-10 (pg/mL) |  |  |  |
| Week 0 | 16.0 (4.8) | 10.9 (2.9) | - 1. (2.9) |
| Week 8 | 16.3 (4.0) | 15.0 (3.2) | 5.2 (2.0) |
| IL-12 (pg/mL) |  |  |  |
| Week 0 | 13.6 (4.9, 27.8) | 5.1 (4.6, 51.2) | - 1. (3.1, 4.7) |
| Week 8 | 17.7 (4.5, 34.8) | 6.4 (4.3, 25.4) | 3.1 (3.1, 5.3) |
| IL-17A (pg/mL) |  |  |  |
| Week 0 | 152.4 (32.9, 683.9) | 83.4 (43.4, 125.7) | - 1. (2.8, 4.4) |
| Week 8 | 182.7 (35.2, 645.9) | 32.25 (8.9, 49.9) | 2.8 (2.8, 3.0) |
| IL-23 (pg/mL) |  |  |  |
| Week 0 | 192.8 (13.3, 684.1) | 38.9 (14.2, 65.4) | 3.5 (3.5, 3.5) |
| Week 8 | 205.1 (15.4, 747.9) | 24.5 (5.8, 54.3) | 3.5 (3.5, 3.5) |

Numbers show mean (standard deviation [SD]) or median (25^th^, 75^th^ percentile)

Abbreviations: ALP, total alkaline phosphatase; PTH, parathyroid hormone; CRP, C-reactive protein, IL-, interleukin-.

**Supplemental Figure 1:** C-reactive protein (CRP) and interleukin (IL-) panel at baseline and week 8 by sub-group.

Line indicates group median. IL-12, IL-17A, IL-23 plotted on log 10 scale for clarity.
